# Using the Ages and Stages Questionnaire to teach medical students developmental assessment: a descriptive analysis

**DOI:** 10.1186/1472-6920-6-29

**Published:** 2006-05-22

**Authors:** Pam Nicol

**Affiliations:** 1 School of Paediatrics and Child Health, University of Western Australia, Princess Margaret Hospital, GPO Box D184, Perth 6840, Australia

## Abstract

**Background:**

After a survey of medical graduates' skills found a lack of confidence in developmental assessment, a program was introduced with the broad aims of increasing medical student confidence and respect for the parents' role in childhood developmental assessment. Research has shown that parents' concerns are as accurate as quality screening tests in assessing development, so the program utilised the Ages and Stages Questionnaire, a parent completed, child development assessment tool.

**Method:**

To evaluate the program, an interpretative analysis was completed on the students' reports written during the program and a questionnaire was administered to the parents to gain their perception of the experience. As well, student confidence levels in assessing growth and development were measured at the end of the paediatric term.

**Results:**

Although there was an increase in student confidence in developmental assessment at the end of the term, it was not statistically significant. However the findings indicated that students gained increased understanding of the process and enhanced recognition of the parental role, and the study suggested there was increased confidence in some students. Parents indicated that they thought they should be involved in the teaching of students.

**Conclusion:**

The ASQ was shown to have been useful in an education program at the level of advanced beginners in developmental assessment.

## Background

Developmental assessment is a core learning outcome for paediatric and child health students, so when a survey of medical graduates' skills identified a lack of confidence in this area, a program was developed with the aims of increasing both confidence and respect for the parental role. This study evaluates that program.

Paediatric and child health practitioners advocate a family-centred care model that requires practitioners to have good interpersonal skills, to have respect for parental judgement and to be flexible in their role [1]. As well, collaborative patient-centred practice is emerging as a framework for interdisciplinary education [2]. For these frameworks to be successful, interpersonal competence, which includes an appreciation of the skill and uniqueness of all individuals involved, is required [3]. The development of these attitudinal and communication attributes in health care practitioners is one of the challenges for health educators interested in family-centred practice.

Medical student attitudes are important because they are viewed as a mediating link between clinical competence (knowledge and skills) and clinical performance and influence what the will do in clinical practice [4]. As students progress through their medical program some attitudes become increasingly negative, for example in relation to doctor-patient relationships, communication and preventative medicine [5]. A graduate survey of final year undergraduate medical students conducted in 2003 in a medical school in Western Australia showed that only 45% of them felt confident in performing a developmental assessment on a child (unpublished data).

Medical educators have suggested, that, to challenge and counter such negative attitudes, curricula could focus on the issue of respect and use structured exercises based on contact between groups as a learning tool [6,7]. Collaboration of parents and clinicians in teaching in medical programs has been shown to be a successful way to improve mutual understanding and effect attitudinal change in the students [8]. A literature review by Wykurz and Kelly (2002) on the role of patients as teachers demonstrated that learners gained important educational benefits from meeting real patients with knowledge and teaching skills who had firsthand experience of a condition. [9]. Studies have shown that patients can successfully deliver tutorials that improve attitudes and skills for medical students [6,10,11].

Parents can help the medical students' learning by suggesting ways to communicate with their child and by providing information on the child's developmental milestones that complements student observations. This adaptive and active view of learning, where students utilise a naturally complex real situation to construct personal knowledge, is grounded in constructivist epistemology [12].

Surveillance of developmental progress is a process of eliciting and attending to parents' concerns, making accurate and informative longitudinal observations on children and obtaining a relevant developmental history [13]. It has been recommended that screening for development should not be limited to inquiry at one point in time and should use tools that have been demonstrated to have adequate psychometric properties [14], but screening tests have not been popular in primary care due to factors such as test length and difficulty managing children's behaviour.

However, research has shown that parents' concerns are as accurate as quality screening tests and that differences in education and child-rearing experiences make little difference in parents' ability to raise important concerns [15]. The Ages and Stages Questionnaire (ASQ) is a parent-completed child development early detection system specifically designed to be part of a child health-monitoring program [16]. However, because it is based on a parent participation model, it was also considered to be appropriate to be used for the structured experiential learning program that was set up in response to the 2003 survey findings to address the lack of confidence in final year students in performing a developmental assessment on a child.

### The program

Approximately 90 students in the fifth year of their medical undergraduate program at a Western Australian university complete an 8-week term in paediatrics and child health each year. During the paediatric term, students are expected to develop skills (to a primary practitioner level) in developmental assessment.

As well as addressing the confidence issue, a secondary aim was to raise the students' respect for the parents' role in developmental surveillance. The program comprises a one-hour lecture on child health and development and a 30-minute tutorial on practical aspects of screening for growth and development, which are followed by an assessment task and a written report. For the assessment task, students measure and chart a child's growth and complete a developmental assessment using two methods; the Stycar developmental milestones to guide the students [17] and the ASQ tool completed by the parents [16].

The Stycar milestones were chosen because they had been used in previous years to guide students' learning, require no formal training and are in the clinical guidelines of the university campus hospital. The ASQ was considered appropriate as an introductory tool for medical students as it is currently being piloted in Western Australia as a population based screening tool, has been validated as a screening tool and does not require specific training. One alternative, the Parents Evaluation of Development (PEDS) tool, which asks 10 short questions of parents, was not chosen as it would contribute less to the development of students' knowledge (and therefore, it was hypothesised, their confidence) than the ASQ [15].

The ASQ is used for developmental screening or monitoring in primary care for ages between 4 and 60 months. Each questionnaire contains 30 developmental items divided into 5 areas: communication, gross motor, fine motor, problem solving, and personal social. An additional overall section addresses general parental concerns. It is scored using the ASQ User's Guide to convert the parents' responses (yes, sometimes or not yet) to points. Referral for further assessment is recommended at a cut off point which is at 2 standard deviations below the mean. It has been well validated on combined risk and non-risk groups; sensitivity 72% and specificity 86% overall ages. There is 94% agreement between parent assessments using the ASQ and expert clinician assessments [16]. Questionnaires usually take between 10 and 15 minutes for a parent to complete.

In the program, students randomly select and obtain permission from a parent waiting for an appointment for a child in the hospital outpatient department. It is not necessary for the child to have developmental problems. If, following the assessment, the ASQ score is below the cut-off score, but the parent is not aware of any problems identified by the student, the child's doctor is requested to review the child at the next appointment. The students complete a reflective report, which is a summary of their findings, their comments on the use of the two tools, and the comparison between their assessment and the parents. There are no names or demographical details of the child or family on the reports. The students are given formative feedback on their report and it does not contribute to the unit's summative assessment.

## Method

### Program evaluation

The aims of the evaluation were to:

1. Determine if the program improved student confidence to assess child development

2. Evaluate student learning from using the two methods of assessment, including their attitude towards the parents' role in assessing their child's development

3. Explore the parents' perception of their involvement in the program.

To measure confidence in developmental assessment, the university's 2004 and 2005 Students Perception of Teaching Surveys (SPOT), were compared with the 2003 SPOT to ascertain whether there had been a change in the mean scores following the program's introduction (Wilcoxon Mann-Whitney Test, p > 0.05). The primary role for SPOT surveys is to encourage ongoing development of teaching and learning and they are administered routinely after each paediatric term. Each item in the questionnaire is rated on a five-point Likert scale (1 = strongly disagree, 5 = strongly agree). One item asks the student to rate their confidence to assess growth and development. This item was used as it would provide a comparison of confidence before and after the program.

Secondly, to evaluate student learning, a phenomenological approach was adopted. An interpretative analysis of a convenience sample of the 52 student reports was completed to saturation, in order to explore student experience. The written comments and reflections of the students were analysed for key concepts and the data were examined for key themes that emerged from the students' comments and reflections. The educator completed the analysis and applied LeVasseur's (p.419) notion of "persistent curiosity' based on technique of bracketing to "suspend prior beliefs" as described by van Mennan (p.175) [18,19]. A similar analytic strategy has been used for evaluation of other teaching programs [8,20].

Finally, the parents were asked by students if they would complete a Likert Scale questionnaire (1 = strongly disagree, 5 = strongly agree) in order to evaluate their perception of their role on the program. The questionnaire was developed using results from an initial review of the student reports and from other teaching programs that have assessed the perspectives of patients as teachers [10,21-23]. The eight questions asked were:

1. a) I found the ASQ easy to complete.

b) How long did it take to complete?

2. When answering the ASQ questionnaire, I felt I was honest about my child's abilities.

3. Participating in this with the student has increased my understanding of my child's development.

4. I felt confident that I know my child best and therefore able to contribute to the student's learning and task.

5. I gained a sense of satisfaction from helping the students.

6. The student was confident in his/her interaction with my child.

7. I found the student respectful to me and my child.

8. Parents should be involved in teaching medical students.

The parents were given a return addressed envelope to return the questionnaire via the departmental clerk. Consent was implied by return of the questionnaire and anonymity was maintained.

### Ethical permission

Ethical permission was obtained from the medical director of the university hospital campus and the hospital ethics committee.

## Results

### Student confidence

The mean SPOT score (Wilcoxon Mann-Whitney Test, p > 0.05) for student confidence in performing a developmental assessment at the end of the terms in which they took the paediatric course in 2003, before the introduction of the program, was 3.95. In 2004 and 2005, after the introduction of the program, it was 4.08 and 4.13 respectively, but the increase in the means was not statistically significant. The response rates were 67%, 86% and 85% respectively.

### Student learning

#### ASQ scores

The findings from the student Stycar assessment and the parents' ASQ report were similar (70% percentage agreement). Where the findings differed, the students sought possible explanations, e.g., some students wrote that the parent explained their child behaved differently at home, an explanation that was usually supported by the ASQ score. The students also noted the busy environment sometimes distracted the child from the assessment task. Further discussion sometimes revealed parental concern rather than actual developmental delay, e.g., one child's parents were concerned about the child's poor pronunciation of words (she had recently had grommets inserted), but the child scored 60/60 on the communication sub-domain of the ASQ and the student assessed there was no language or communication deficit. At other times, the student's assessment appeared inconsistent with the ASQ findings, e.g. one child had a speech and language delay on ASQ (the child's problem solving was borderline, there were no intelligible words, no recognition of pictures, tended to ignore the mother) but the student found the child's hearing normal.

#### Key themes

There were five key themes arising from an analysis of the students' reports. These were:

##### 1. Increased understanding of the process of screening for development

The students' comments demonstrated that through use of the ASQ compared to using the Stycar alone, the exercise increased their understanding of the range of normal development and what was considered an acceptable cut off score, as well as their understanding of parents' perceptions or feelings, e.g., that the parents were happy to know their child's development was similar to their peers and what parents considered were normal milestones. Through interaction with parents structured around the ASQ, there was increased reporting as compared to previous student assessments in earlier cohorts of play, socio-emotional and problem solving. Students often prefaced assessment with good observational descriptors of the child's temperament and interaction.

"A happy and interactive child who initially engaged with me got tired and bored and turned to mother for a hug."

The two methods were both generally considered to be useful, but with limitations such as operator skill and the child's cooperation. Comments about using the ASQ as a tool indicated it was more helpful than the Stycar guidelines, because it was clearly set out and age-specific, it allowed for graded responses and gave specific examples, and it was easier to undertake, simple to interpret, and useful if the parent had limited English.

"Now understand I can do a valid and valuable appraisal using simple tools."

"The structured approach of ASQ enabled me to direct my attention."

"The ASQ gave me a better mental picture."

"The ASQ helps the parents give a more unbiased opinion regarding their child."

##### 2. Awareness of techniques and engagement of the child

Student comments revealed they gained a lot from interaction with the child. They learnt about: observing the child at play; the challenge of creating rapport before attempting the assessment; the need for flexibility of tasks; the importance of preparation; using play as a test tool; the realisation that how you say things can influence the reply.

"They will naturally do a lot of tasks you want them to do."

"I was interested to see trust develop in the child, happy to be weighed without mother despite very shy at first."

"I took a non-structured approach as I soon realised this would be impossible."

"I learnt not to play ball games before less exciting activities."

"No chance to stop and read through milestones while a very active toddler is constantly running away to go and play with other kids."

"Easy to structure the play to test specific components."

##### 3. Increased confidence in recognition of normal development and problems

Only one student commented that the exercise was not useful for learning. All the others stated that the exercise increased their confidence in assessment of development.

"Despite initial hesitations, this has been useful exercise; actually doing has cemented the ideas more firmly in my mind and increased my confidence in assessing and interpretation."

"I can now explain results more succinctly."

##### 4. Parents as reliable and valid reporters

Two sub themes emerged:

a) Recognition of parents as expert in their children, as they spend the most time with them.

"I found the child hard to engage verbally, he rarely followed instructions and generally ignored the test administrator. I felt comfortable accepting the parent's input, as they were more used to his speech and mannerisms, in this way, I felt I gained a more accurate picture of his communicative development."

b) Potential parental bias. Some students commented on the parents' ability to complete a reliable and valid assessment of their child. In some cases this was appropriate, e.g., when the parents expressed concern about their child's diminished visual acuity following cataract surgery, although no gross visual deficit was observed and the ASQ was well above cut off for vision. However, some comments revealed student perceptions that parents could over-estimate their child's ability rather than give an objective assessment. This was even the case when the ASQ demonstrated good concordance with the student's assessment.

"(The) mother seemed to have a good grasp on the developmental level; I understand this may not be so in many other mothers, especially those with developmentally delayed children."

"Even though ASQ more specific, I still think observer's examination more useful, mother may have bias or mislead the examiner, whilst physical examination allows clinicians to identify problems without mother's bias affecting the result. ASQ can fill in the gaps, which may be true at home."

"The mother may have overemphasised her child's communication ability and seemed intent on making him do what was requested, even interrupting his play, perhaps I needed to clarify any concerns the mother had."

##### 5. Enjoyment of the process

The students enjoyed the assessment and often completed further assessments independently.

"I enjoyed the opportunity to perform this assessment, because in many ways it relies on actually joining in with the child's play, something that I always enjoy given the chance!"

"I would benefit from doing more of these with children of all different ages because it really cements in my mind the abilities the children have at different ages."

### Parents' perception of their role

The response rate for the parents' questionnaire was 61% (N = 31). All parents found the ASQ very easy or easy to use (mean 4.2) and took between 5 and 45 minutes (mean 13 minutes) to complete it. The students were considered respectful (mean 4.64) and fairly confident in approaching the child (mean 4.5). Parents felt confident they knew their child best and perceived they were therefore able to contribute to the students' learning (mean 4.4). However one parent was concerned that students relied too heavily on their assessment. Overall the parents felt they learnt more about their child by participating, but this was the weakest response (mean 3.9). All agreed they were honest in describing their child's abilities (mean 4.6). They also agreed that parents should be involved in teaching students (mean 4.3) and said they gained a sense of satisfaction from participating (mean 4.4). Some examples of their comments:

"I feel parents have a role in providing anecdotal evidence and in raising issues they would like to know more about (what they would like doctors to be able to answer)."

"It was a good idea to do the questionnaire with the student. As a parent I am the one most able to answer the questions about my child."

"I was very impressed with the student's professional manner but also with their caring and friendly attitude."

"I was happy to help out and will continue to do so in the future if asked to."

"I think the student should give themselves more time to assess the children independently. As some parents may be a little biased toward their child's development, they should assess on what they see."

## Discussion

There is a difference between the quantitative and qualitative outcomes. This is probably because they are largely addressing different things. The SPOT scores directly addressed confidence and indicated there was no significant change resulting from the program. However the qualitative research, based on student comments that were not directed, tended to be directed more towards understanding than confidence. For example, the comments indicated that the program expanded students' understanding of developmental milestones and of the two methods of assessment, as well as how to play with children and interact with their families. There was also an increased recognition of parents as partners in the assessment process.

Unfortunately, there were no examinations in the course in this area until 2005, so a more objective indication of the effect of the program on understanding was not available.

Where students commented on confidence, all but one commented that their confidence had increased. This appeared to be in contrast to the SPOT findings. However the finding is congruent with a study on teaching communication skills to medical students using a formal structured approach that found a decrease in students' confidence following the program. The authors attributed this to reflexivity and introspection in the program leading over confident students to reassess their skills and commented that under confident students were offered a model which they could use for practice [24]. From this, it may be surmised that a proportion of students in the study reported in this paper could have lost confidence but not commented on this their reports. This is supported by some students' completing more than the one required assessment recognising the differences with age of the child. If this were the case, it would not be apparent until the SPOT scores were available. Confidence could also be affected by the time delay between the writing of the report and the SPOT survey at the end of the term. Alternatively, although the students' confidence may have increased qualitatively, this may have been insufficient to lead them to choose 'strongly agree' over 'agree' in the SPOT Likert Scale.

The results cast some doubt on the underlying assumption in using the ASQ that increased knowledge would translate into increased confidence. Confidence is a complex attitude, of which knowledge is only one component. In the case of beginning practitioners, how many times and in what context a skill is attempted will influence confidence. This program deliberately placed the student in an unpredictable real-world context in order to learn about the process and to value parents' input. As students became aware of the complexities of assessing child development it is possible their confidence would be less robust. Some students commented that more of these assessments over all age groups would help increase their confidence. This makes intuitive sense from clinical experience. Correlation of objective assessment of knowledge and skill in a structured clinical exam with confidence and attitude would be of interest for future research.

Awareness of the importance of play and the use of structured validated tools is a useful outcome for future practice. The students preferred using the more structured ASQ tool as it provided better guidance than the Stycar milestones. This was appropriate for their level of competence as advanced beginners; the tool itself became part of the learning. The parent helped students relate to the child and complete a skill that may have been beyond their ability without support. This is the process of scaffolding that is central to constructivist theory, the theoretical base for the program [25]. Experiential learning requires structure, and the complexities of the skill being learnt require the components being made clear to the learner [26]. This is sometimes overlooked in apprenticeship style learning in clinical practice.

A disadvantage of a student centred program is decreased control over the learning that occurs. The results showed a 70% agreement between student and parent. This was lower than the 94% reported in ASQ reliability studies. The most likely explanation for the difference was student lack of experience: the ASQ score supported the parents' assessments over the students in many (but not all) cases. Despite this difference, or more likely because of this difference, most students acknowledged the important role parents play in developmental assessment, even though a few students continued to believe that parents may overestimate their child's abilities. Not surprisingly, parents did not feel they overestimated their child's abilities. Follow up discussion with tutors and peers to challenge the students' attitudes and reflect on their experience would be useful to address differences in perception [26]. Student discussion groups to facilitate reflection and self evaluation of learning was found to be useful in helping raise students awareness of patient-centred medical interviewing techniques in a study which aimed to compare student and maternal evaluations of simulated interviews [27].

The student comments suggest the experiential nature of the program creates more effective learning than a more didactic approach to teaching developmental assessment. The process of learning and concept development can be powerful outcomes in themselves. This illustrates double-loop learning, where the activity is part of a larger cycle, in which the reflection takes place by engaging in the activity in a real world context [28]. When there was a difference in ASQ assessment results, the students reflected on the situation and used a problem solving approach to identify what may have been real concerns for the mother; the process of 'reflecting-on-action' [29].

A limitation of the study is the use of reflective writing, especially for assessment, which can be criticised for bias in that students may write what they know the reader – in this case the educator – wants to read. However students often do more than the one required assessment and are quite animated after doing the assessment, recounting stories from their experience to the educator. The study would be more robust if a validated attitudinal test had been used, but this was not possible for this study. It would however, be a useful future study, as there is little in the literature about student attitudes to parent involvement in developmental assessment.

In this study, parents agreed they should be involved and felt the students benefited from their input. Future research could explore the effect on attitudes, knowledge and practice if parents were trained as tutors to teach developmental assessment. This knowledge could help direct future program development and evaluation.

## Conclusion

The evaluation of the program has indicated that the ASQ can be an appropriate tool for students at the level of advanced beginners in paediatric developmental assessment. Despite contrasting research results on student confidence levels, students reported increased understanding and were positive about the program. The long-term benefits of the interpersonal skills and attitudes that they learn may be more important than confidence in the actual skill at this stage of their careers.

## Competing interests

The author(s) declare that they have no competing interests.

## Authors' contributions

The contributing author was the sole designer of the program, the program evaluation and the writing of the manuscript, and was the educator for the program.

## Pre-publication history

The pre-publication history for this paper can be accessed here:


